# Supplementary material for: The interactome of intact mitochondria by cross-linking mass spectrometry provides evidence for coexisting respiratory supercomplexes
Source: Mol Cell Proteomics. 2017 Dec 8;17(2):216–32. doi: 10.1074/mcp.RA117.000470 (PMC5795388; doi:10.1074/mcp.RA117.000470)
Supplement: Supplemental Data [file supp_RA117.000470_133922_0_supp_23978_fzffwf.zip › spectra_annotation/mito_spectra_annotation/193-1-1-1-7-1.pdf]

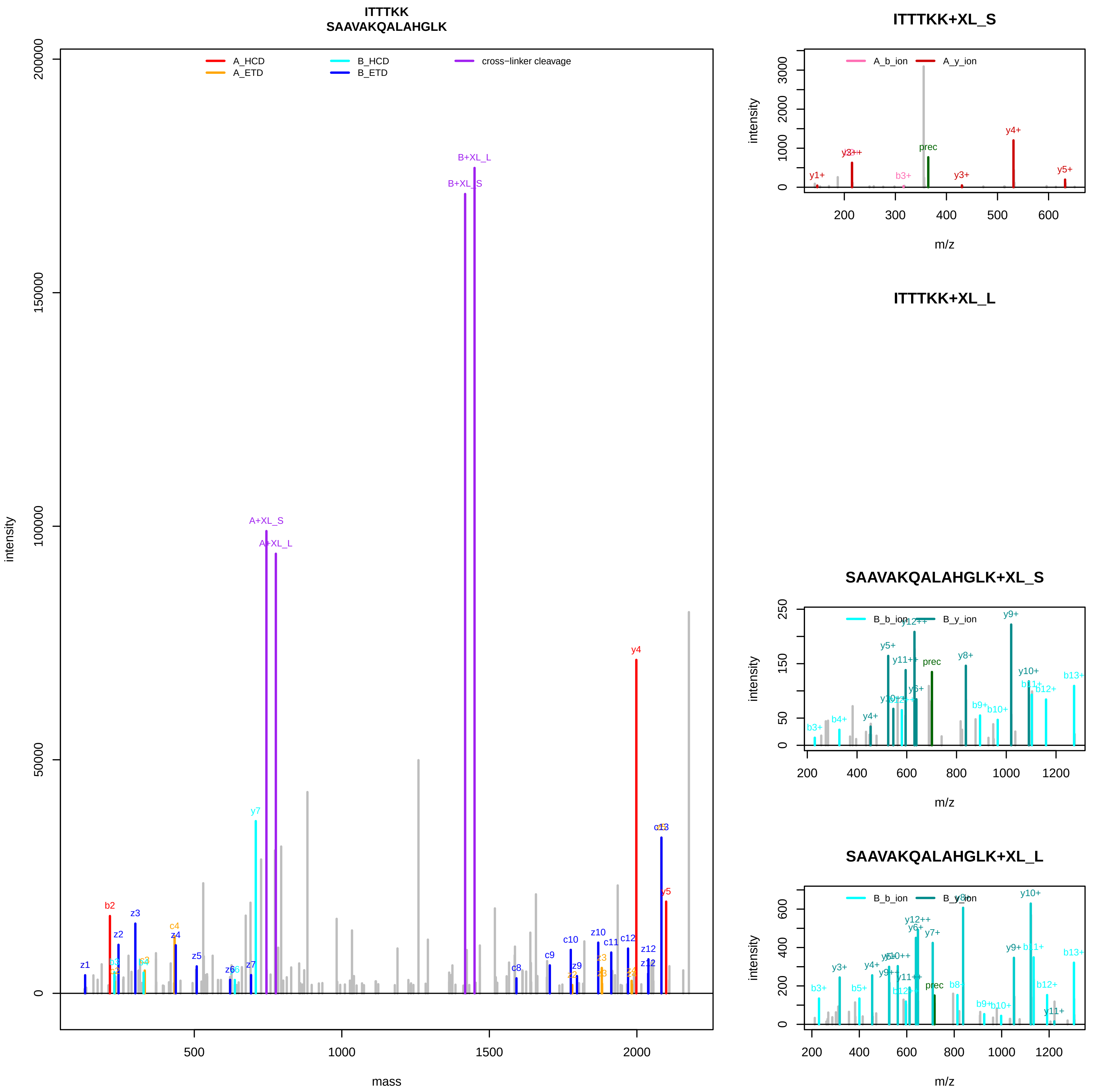
ITTTKK
SAAVAKQALAHGLK
A_HCD
A_ETD
B_HCD
B_ETD
cross−linker cleavage
intensity
mass
B+XL_L
B+XL_S
A+XL_S
A+XL_L
y4
y5
y7
c13
b2
z3
z2
z4
c4
z5
z6
z7
c8
c9
c10
z9
z10
c11
c12
z12
ITTTKK+XL_S
A_b_ion
A_y_ion
y1+
y3++
b3+
prec
y3+
y4+
y5+
m/z
ITTTKK+XL_L
SAAVAKQALAHGLK+XL_S
B_b_ion
B_y_ion
SAAVAKQALAHGLK+XL_L
